# Structural equation modeling of psychosocial determinants of health for the empowerment of Iranian women in reproductive decision making

**DOI:** 10.1186/s12905-020-0893-0

**Published:** 2020-02-03

**Authors:** Zahra Kiani, Masoumeh Simbar, Mahrokh Dolatian, Farid Zayeri

**Affiliations:** 1 Student Research Committee, School of Nursing and Midwifery, Shahid Beheshti University of Medical Sciences, Tehran, Iran; 2 Midwifery and Reproductive Health Research Center, Shahid Beheshti University of Medical Sciences, Tehran, Iran; 3 Department of Midwifery and Reproductive Health, School of Nursing and Midwifery, Shahid Beheshti University of Medical Sciences, Tehran, Iran; 4 Proteomics Research Center and Department of Biostatistics, Faculty of Paramedical Sciences, Shahid Beheshti University of Medical Sciences, Tehran, Iran

**Keywords:** Decision making, Reproductive, Social determinants of health

## Abstract

**Background:**

Women’s empowerment is a process wherein females are afforded power over their own lives as well as their participation in the communities and larger societies to which they belong. An important aspect of such empowerment is the right to make decisions regarding fertility—an entitlement affected by the social health determinants that contribute to the social conditions under which humans live and work throughout their lives. As one such determinant, psychosocial factors play an essential role in the development of women’s empowerment. Correspondingly, this study conducted a structural equation modeling of these determinants to examine the empowerment of Iranian women in reproductive decision making.

**Methods:**

This cross-sectional study involved 400 women who were referred to clinical centers of the Shahid Beheshti University of Medical Sciences in Tehran, Iran. Data were collected using six questionnaires, namely, demographic, socioeconomic, and social support questionnaires, the Rosenberg self-esteem scale, a marital satisfaction questionnaire, and an empowerment survey. The data were analyzed using SPSS software version 17, and the structural equation modeling was carried out using EQS software version 6.1.

**Results:**

The Iranian women had an average level of empowerment with respect to reproductive decision making, and such empowerment was related to all the psychosocial factors examined (*p* = 0.001). The final model appropriately fit the data (comparative fit index = 0.92, root mean square error of approximation = 0.06). The psychosocial factors served as intermediate social determinants of the women’s empowerment in reproductive decision making (β = 0.78, *p* = 0.001). This empowerment was indirectly affected by socioeconomic situation as a structural factor (β = 0.44, *p* = 0.001).

**Conclusions:**

Socioeconomic factors, through the mechanism of psychosocial determinants, may significantly affect women’s empowerment in making decisions regarding reproductive health. Conditions associated with these factors should be improved to ensure that women claim and exercise their right to have mastery over their reproductive health.

## Background

Sexual and reproductive health and the rights associated with these are critical factors for empowering women and advancing gender equality. Such equality depends centrally on giving women the freedom to exercise their right to make free and informed choices about their sexual and reproductive lives and whether and when to have children [1]. Empowerment, which refers to the ability to make independent decisions in different situations, including those related to health concerns, is recognized as a personal entitlement [2]. Empowering vulnerable populations thus requires special attention [3].

The 2011 statistical report of the World Health Organization (WHO) indicated that in developing countries, 12% of 15- to 49-year-old women do not use contraception despite their desire to do so, which means that nearly 222 million females in these regions forgo birth control and are thus exposed to the risk of unwanted pregnancies [4]. Many WHO programs emphasize reproductive health and its promotion through women’s empowerment with respect to reproductive decision making [5]. Given that health and well-being arise from the individual exercise of power, women’s empowerment exerts positive effects on their health and quality of life and those of their families [6]. Such empowerment is the process in which women elaborate and recreate what it is that they can be, do, and accomplish in circumstances that they were previously denied [7]. It is considerably influenced by the environment in which people are born, live, and work—a set of conditions collectively referred to as social determinants of health. The WHO recognized the impact of social determinants on health in 1948 and underscored the development of appropriate health strategies and the provision of primary health care for all people in the Alma-Ata Declaration of 1978 [8]. On the basis of the WHO’s model of social health determinants two categories of factors were defined: structural determinants, which encompass gender, income, education, occupation, social class, and race / ethnicity, and intermediate determinants, which cover environmental, psychosocial, and behavioral factors. Structural determinants are those that generate or reinforce social stratification in the society and that define individual socioeconomic position. These mechanisms configure the health opportunities of social groups based on their placement within hierarchies of power, prestige and access to resources (economic status). Psychosocial factors are especially influential on quality of life, receipt of care, and effective functioning or work. Psychosocial factors includes Psychosocial stressors, stressful living circumstances and relationship, and social support and coping style (or the lack thereof) [4, 9].

The social, environmental, and cultural aspects of social health determinants directly affect the well-being of a population [10]. They are the health-promoting factors that exist in living and working conditions (e.g., the distribution of income, wealth, influence, and power) rather than individual risk factors that influence the possibility of disease occurrence or vulnerability to illness or injury (e.g., behavioral risk factors or genetics) [11]. Social determinants of health are critical factors for achieving health equality [4]. Accordingly, the Cairo Conference emphasized the reduction of gender inequality fundamentally through the empowerment of vulnerable groups either at the individual or at the societal level [12]. With regard to women, in particular, this empowerment is enabled by their education, occupations, and economic conditions [13, 14]. Improving their socioeconomic conditions, in turn, cultivates their capacity to insist on the right to engage in reproductive decision making [15]. Unfortunately, women do not use contraceptives given the lack of or weak support from spouses or communities [16]. Their empowerment in this regard is also particularly influenced by their ability to communicate with their spouses [17, 18].

In reviewing the literature, factors such as the education of women and their husbands’ [14, 19, 20], the occupation of women and their husbands’ [14, 20–22], asset index [3, 4], self-confidence [4, 23], marital satisfaction [14, 24] and social support [17, 25] have been associated with women’s empowerment in reproductive decision making. On the other hand, the World Health Organization model has identified these factors as determinant Social health is divided into two groups: structural (occupation, education and asset index) and intermediate (self-confidence, marital satisfaction, social support).

The empowerment of women in formulating and implementing reproductive health-related decisions has been modeled in different ways. Leininger, for example, modeled reproductive health care provision as an important influencing factor for reproductive decision making, which in turn, is affected by social conditions [26]. Mahmoud et al. developed a conceptual model wherein women’s decision-making empowerment and self-esteem are linked to their social statuses [19]. In our literature review, we found no related article about the relationship between social determinants of health (structural and intermediate determinants) and women’s empowerment in reproductive decision making in the Iranian population. While, providing the model of women empowerment in reproductive health can lead to a comprehensive plan to improve women’s ability for making informed decisions in their sexual reproductive health.

### Objectives

Considering the role of social health determinants, especially psychosocial ones, in women’s empowerment in reproductive decision making, the present research performed a structural equation modeling (SEM) of these factors to inquire into women’s empowerment in reproductive decision making in Iran. The analysis was based on the WHO’s model of social determinants of health.

### Hypotheses

The Socioeconomic status (Including: occupation, education and asset index) and psychosocial factors (Including: self-confidence, marital satisfaction, social support) have positive and significant relationship with women’s empowerment in reproductive decision making. Psychological factors directly affect women’s empowerment, while economic factors indirectly affect women’s empowerment.

## Methods

### Study design and sample

This cross-sectional study involved 400 women who were selected in 2014 via multistage cluster sampling from attendees of health centers of the Shahid Beheshti University of Medical Sciences in Tehran, Iran. In sampling process, we first listed all 54 health centers of this university and then randomly selected 12 centers. Finally, using a probability proportional to size (PPS) sampling method, the sample participants were recruited from the selected centers. The inclusion criteria were an age range of 15 to 49 years, being literate, living in Tehran for more than a year, being of Iranian nationality and Muslim descent, having no history of depression, being of good mental health, being married and currently living with husband, being a husband’s only wife, having at least one child, having no history of infertility and relevant treatments, and currently not undergoing pregnancy.

### Sample size

**Fig. 1:**
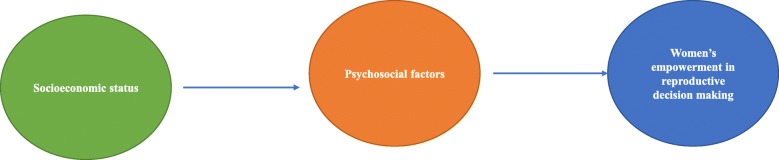
Conceptual framework

**Fig. 2:**
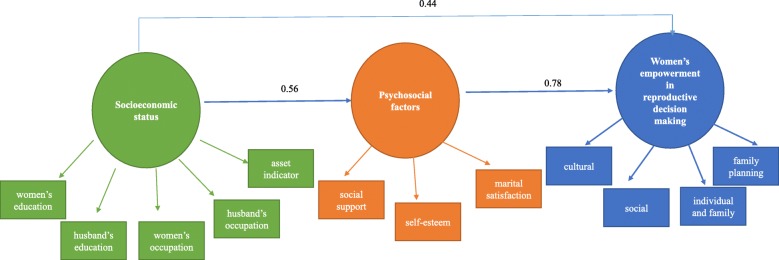
Structural Equation Model of Psychosocial social determinants of health for women’s empowerment in reproductive decisions

Regarding our literature review about the sample size determination in SEM, a sample of 200–400 is recommended for maximum likelihood (ML) estimation of multivariate normal data based on Monte-Carlo investigations [27, 28]. Mplus software version 7.0 was used and at least 5:1 ratio of cases of free parameters. The parameter and standard error biases do not exceed 10% for any parameter in the model (Figs. 1 and 2). The standard error bias for the parameter for which power is being assessed does not exceed 5%. The coverage remains between 0.91 and 0.98. A power of 0.8 was used as the commonly accepted value for sufficient power of test. Regarding this, a sample size of 400 was calculated.

#### Demographic questionnaire

A demographic questionnaire was administered to the participants to determine their ages, histories of gravid/para and planned pregnancies, number of abortions, the ages in which they entered into marriage, their ages at the birth of their first children and the genders of these children, the total number of children that they have, and income.

#### Socioeconomic questionnaire

The socioeconomic questionnaire was used to assess the socioeconomic statuses of the participants. The number of years during which they attended schooling was used to examine educational status, and the asset indicator [e.g., ownership of a vacuum cleaner, separate kitchen, computer, washing machine, bathroom, freezer, dishwasher, private car (not used for work), mobile phone, color TV, various types of video and telephone equipment] was used to assess economic status [29]. The asset indicator was then converted into percentage form. The occupational statuses of the women and their husbands were classified following the method of Ross et al. [30], leaving us with 17 main categories, each with subcategories that in total corresponded to 30 occupations.

#### Perceived social support questionnaire

The participants were asked to complete the multidimensional scale of perceived social support (MSPSS) [31], which contains 12 items regarding Family, friends and important people, among other support-related issues. Its validity and reliability have been reported as being of appropriate levels [31–33]. The internal consistency (IC) of the perceived social support questionnaire was assessed using the Cronbach’s alpha. The obtained value of 0.92 showed an acceptable IC for this instrument.

#### Rosenberg’s self-esteem scale

Rosenberg’s self-esteem scale is a standard questionnaire with 10 items. It is recognized as a valid instrument, with a reliability of 0.85, as determined through test–retest reliability assessment [34]. IC of the perceived social support questionnaire was assessed using the Cronbach’s alpha. The obtained value of 0.73 showed an acceptable IC for this instrument.

#### Marital satisfaction questionnaire

The marital satisfaction questionnaire measures marital problems, which are reflected in 25 questions. Its validity and reliability have been assessed and confirmed [35, 36].

IC of the perceived social support questionnaire was assessed using the Cronbach’s alpha. The obtained value of 0.94 showed an acceptable IC for this instrument.

#### Women’s empowerment in reproductive decision making questionnaire

The women’s empowerment in reproductive decision making (WERD) questionnaire [37] is intended to evaluate women’s ability to make decisions about reproduction. It consists of 38 questions, to which responses are to be indicated through a five-point Likert scale (0 = “strongly disagree,” 1 = “disagree”, 2 = “no idea”, 3 = “agree” and 4 = “strongly agree”). that covers four dimensions: cultural (11 questions), individual and family (10 questions), social (9 questions), and family planning (8 questions). Mean scores are calculated for the entire questionnaire and for each of the four dimensions, and then these are converted into percentages. Finally, the scores are categorized into five classes, namely, 0 to 20, which denotes very weak empowerment; 21 to 40, which signifies weak empowerment; 41 to 60, which indicates moderate empowerment; 61 to 80, which reflects good empowerment; and 81 to 100, which represents very good empowerment.

The content validity ratio (CVR) and content validity index (CVI) of the WERD questionnaire was also assessed by 10 experts. As indicated in Lawshe’s table, a CVR of 0.62 or higher points to acceptable validity [38]. The CVI was examined on the basis of Waltz and Bausell’s criteria, after which the relevance, clarity, and simplicity of each item in the instrument were determined [39]. The evaluation of relevance revealed a mean of 0.96, and the assessment of simplicity and clarity yielded mean values of 0.93 and 0.94, respectively. To ascertain the reliability of the questionnaire, the research conducted test–retest assessment, which yielded a correlation of 0.77. The Cronbach’s alpha coefficient (≥0.70) also confirmed the acceptability of the entire questionnaire and its four dimensions [40].

### Statistical analysis

This study was designed on the basis of the conceptual model to assess the relationship between **structural determinants** [socioeconomic status (a **latent variable**): observable variables such as education, occupation, and asset indicator] and **intermediate determinants** [psychosocial factors (**latent variables**): observable variables such as self-esteem, social support, and marital satisfaction], and concurrently to examine the relationship between psychosocial factors Women’s empowerment in reproductive decision making. Women’s empowerment in reproductive decision making is also a latent variable with observable variables as its dimensions. Fig. 1 shows the conceptual framework.

The Structural Equational Modeling (SEM) approach was utilized to assess the relationship between the described variables according to the conceptual framework in Fig. 1. The usual Fit indices for evaluating the model goodness of fit (RMSEA, CFI, GFI, NNFI, AGFI and χ^2^/df) [41] were also reported. The SPSS software version 17.0 and EQS software version 6.1 were used for data analysis. *P*-values less than 0.05 were considered statistically significant.

## Results

The Mean ± SD age of the women was 31.10 ± 6.50 years. Almost half of them (48.8%) experienced their first pregnancies and many of them (76.5%) had gotten married when they were 18 to 28 years old (Table 1).

**Table 1:** Demographic and reproductive characteristics of women referred to health centers of Shahid Beheshti University of Medical Sciences

Demographic and reproductive characteristics	Mean	SD
Age (year)	31.10	6.50
Age at marriage (year)	25.52	2.5
Age at first childbirth	27.42	3.5
Asset index (percent)	80.21	15.13
	Number	Percentage
Number of deliveries		
1	192	48.0
2	157	39.2
3	45	11.2
≤ 4	6	1.6
Number of children		
1	195	48.8
2	158	39.5
3	40	10.0
≤ 4	7	1.7
Number of abortion(s)		
-	303	75.8
Yes	97	24.2
Children’s gender		
Female	145	36.2
Male	146	36.5
Female and male	109	27.2
Unplanned pregnancy		
Yes	95	23.7
No	305	76.3
Employment status		
Housewife	352	88.2
Employed	48	11.8
Women’s educational level		
Elementary school	39	9.8
Guidance school	78	19.5
High school	180	45.0
Associate degree	36	9.0
Bachelor	67	16.7

The Mean ± SD score of the women’s empowerment in reproductive decision making was 82.54 ± 14.00 of the total score of 152; that is, they obtained 54.3% (moderate) of the total score. The highest score was obtained in the cultural domain (63.2%, good empowerment), whereas the lowest score was derived in the family planning domain (34.7%, weak empowerment). The descriptive statistics of the intermediate and structural determinants of health are presented in Table 2. The data on occupational classification suggested that most of the women fell under the subcategory “not classifiable for other reasons (L17)” and that most of the husbands fell under the subcategory “semi-routine technical occupations (L12-3)” [see, e.g., Ross et al. [23]].

**Table 2:** Descriptive statistics of structural and intermediate social determinants of health

Variable	Mean	Minimum	Maximum
Women’s education (years)	11.40 ± 3.30	2	19
Men’s education (years)	11.30 ± 3.57	4	23
Asset indicator (%)	80.21 ± 15.13	8	100
Self-esteem	29.04 ± 3.30	17	40
Multidimensional perceived social support	60.46 ± 12.48	12	84
Marital satisfaction	44.43 ± 14.46	17	85

The correlation between the structural and intermediate social determinants of health and the participating women’s empowerment in reproductive decision making was evaluated before SEM was performed. The results showed that all the variables were correlated with the women’s empowerment (Table 3).

**Table 3:** Correlation between structural and intermediate social determinants of health and women’s empowerment in reproductive decision making

Variable	Women’s empowerment in reproductive decision making
Women’s empowerment in reproductive decision making	1
Women’s education	0.44**
Men’s education	0.36**
Women’s occupation	0.22**
Men’s occupation	0.29**
Asset indicator	0.39**
Self-esteem	0.34**
Social support	0.32**
Marital satisfaction	−0.34**

*All values are significant at the 0.05 level

**All values are significant at the 0.001 level

Data distribution was normal, as indicated by the Kolmogorov–Smirnov test. In the original tested model, all paths were significant with respect to women’s empowerment in reproductive decision making (Fig. 2). The findings also reflected that the model reasonably fit the data (RMSEA < 0.08, CFI = .092, GFI = 0.94, χ^2^/df = 2.82) (Table 4).

**Table 4:** Model fit indices

Goodness of fit	χ^2^	χ^2^/df	RMSEA	CFI	GFI	AGFI	NNFI
	146.64	2.82	0.068 [0.055–0.080]	0.92	0.94	0.91	0.90

The psychosocial factors directly influenced the women’s empowerment with regard to reproductive decisions (β = 0.78), whereas socioeconomic status indirectly affected such empowerment (β = 0.44). These effects were significant (*p* = 0.001), and the model explained 0.61% of the dispersion in the women’s power to engage in reproductive decision making.

## Discussion

This was the first study that provides a model of women’s empowerment in reproductive health decision making. This model shows the relationship between psychosocial determinants of health with women’s power to exercise the right to reproductive decision making in Iran. In our knowledge, there is not any comprehensive model of psychosocial determinants of women’s empowerment in reproductive- health related decision making, that we are providing for the first time in the present study. Although this model could be applicable for all women, however, the level of relationship between the Psychosocial factors with women’s power for making reproductive health related decisions may be different in various communities. This model helps to make appropriate base for making a model based interventions for women’s reproductive health related decisions.

The results indicated that the women had an intermediate level of empowerment, with the lowest observed in the family planning dimension. The relationship between women’s empowerment and implications for fertility is very complicated. Some family planning behaviors were examined as empowerment indicators that are prominently related to family planning outcomes, family planning, or upcoming objectives [16]. Kohan et al. found that women’s access to sources of information about family planning, their authority in making decisions, and their decision-making skills are the primary determinants of reproductive decision making. The authors concluded that current family planning programs are inadequate for satisfying the reproductive needs and desires of women [30]. Note, however, that the empowerment of women is a complex issue because, first, power and empowerment are intricate and multidimensional concepts that are difficult to acquire, and second, not all norms and behaviors can be considered criteria for empowerment. As regards the latter, the manner by which people conduct themselves and the conventions to which they adhere affect empowerment in diverse ways in different societies; these also change in certain societies over time [15, 42].

The findings revealed that all the structural factors (occupation, education, and income) were related to the women’s empowerment in reproductive decision making. Empowerment can be represented by three basic ideas: right, choice, and process. In the decision-making process, demographic characteristics, women’s education, spouse’s education, occupation, and property present the potential to influence informed decision making. This process can also be affected by characteristics such as religion, place of origin, and cultural practices [14]. Because structural determinants refer to political, social, and economic issues, they also cover social and economic statuses [14, 43], which influence the use of health facilities [44]. Employment status, education level, and income are the most important indicators [45], which were also introduced at the Cairo Conference as facilitators of empowering women in health issues [46]. Whereas education is an essential principle of women’s empowerment, social and cultural norms are considered obstacles to its realization [47]. In fact, structural determinants affect women’s empowerment through intermediate determinants that include psychosocial components as the most important factor [4].

The results likewise demonstrated that problems in the relationship between men and women are associated with women’s empowerment in decision making about reproduction. As discovered by D’Souza et al. [17], marital relations influence reproductive decision making, and women’s poor health and relations with their spouses lead to low-quality marriages. Hindin [48] found a significant relationship between women’s reproductive health and the involvement of men in this issue; encouraging them to participate in resolving reproductive problems is an important strategy for empowering women. The distribution of power between men and women is affected mainly by the gender roles in family and society. Gender is a social construct that describes a set of characteristics, roles, and patterns of behavior that affect one’s ability to decide on the distribution of power and influence in all aspects of marital life [49, 50]. Additionally, marital relationships are effective avenues in which to improve reproductive and women’s health [17, 51]. Nevertheless, contradictory findings have been presented in this regard, with some studies showing that increased empowerment of women through appropriate spousal communication elevates the risk of failure to satisfy needs. This shows that while empowering in one area may be sufficient to meet demand and eliminate barriers, in other dimensions, this may create false confidence in pregnancy prevention measures without the aid of family planning methods [16].

The current research suggested that self-esteem was related to the women’s empowerment in terms of ministering to their reproductive health. Similarly, Mahmud et al. and Sujatha and Reddy showed that women’s independence is a key factor for achieving their reproductive desires and afford them the freedom to plan for childbearing [19, 23]. Empowering women means greater female access to resources and control over their lives, which foster independence and self-esteem and enhances their attitudes about themselves [52]. Self-esteem and empowerment are effective on their reproductive behaviors [53].

Another finding of note in this work is the relationship between social support and the participating women’s empowerment in reproductive decision making. Social support is a coping mechanism and a psychological resource that stems from positive relationships as an agent that empowers women. Traditionally, empowerment is defined by an individual’s ability to control his/her life and obtain supportive resources for goal attainment. Empowerment is a cyclic and interpersonal process that progresses through collective discourse [54]. Social support also considerably contributes to health. Note, however, that the practical and emotional social support that people receive varies depending on socioeconomic status [9]. As reported by Kariman et al., social support affects decision making regarding having the first child [24]. Another study identified social support as a driver of healthy behaviors and a factor that exerts a strong protective effect on health [55]. The lack of adequate support for women leads to the loss of job opportunities and reduced participation in the community [56].

The model proposed in the present research differs from those put forward by Leininger [26] and Mahmoud et al. [19] in that the latter regarded empowerment only as a health outcome, whereas the current work examined effective empowerment factors on the basis of the WHO’s model of social determinants of health. These factors were also divided into structural and intermediate determinants, thus ensuring significant fit in the prediction of structural and intermediate factors that influence women’s empowerment in reproductive decision making. Our model also indicated that 61% of the women are empowered in such decisions and that socioeconomic factors, through psychosocial determinants, may influence their reproductive health-related decisions.

### Limitations

The cross-sectional design of the present research was a limitation of study as direction of causality of the relationship may be questionable in this study. Another limitation is that the results are generalizable to the Iranian community and countries characterized by similar psychosocial conditions. The model should be tested for applicability to other communities.

## Conclusion

Empowering women is a challenging task because its structure is hidden, and no consensus has been achieved as to its exact definition. Empowerment should be defined in cultural terms specific to each society. Women’s empowerment in reproductive decision making is a complex issue, but some of its important psychosocial determinants were explored in this study. Conditions related to these factors should be improved to ensure that women are guaranteed the right to engage in reproductive decision making.

## Data Availability

The datasets used and/or analyzed during the current study available from the corresponding author on reasonable request.

## Abbreviations

AGFI: Adjusted goodness of fit index
CFI: Comparative fit index
CVI: Content validity index
CVR: Content validity ratio
GFI: Goodness of fit indices
IC: Internal consistency
ML: Maximum likelihood
MSPSS: Multidimensional scale of perceived social support
NNFI: Non-normed fit index
PPS: Probability proportional to size
RMSEA: Root mean square error of approximation
SEM: Structural Equational Modeling
WERD: Women’s empowerment in reproductive decision making
WHO: World Health Organization
